# Mental rotation performance in soccer players and gymnasts in an object-based mental rotation task

**DOI:** 10.2478/v10053-008-0135-8

**Published:** 2013-06-17

**Authors:** Petra Jansen, Jennifer Lehmann

**Affiliations:** Institute of Sport Science, University of Regensburg, Germany

**Keywords:** embodied cognition, motor expertise, gender effect, rotational experts

## Abstract

In this study, the effect of motor expertise on an object-based mental rotation task was investigated. 60 males and 60 females (40 soccer players, 40 gymnasts, and 40 non-athletes, equivalent males and females in each group) solved a psychometric mental rotation task with both cube and human figures. The results revealed that all participants had a higher mental rotation accuracy for human figures compared to cubed figures, that the gender difference was reduced with human figures, and that gymnasts demonstrated a better mental rotation performance than non-athletes. The results are discussed against the background of the existing literature on motor experts, mental rotation performance as well as the importance of the testing situation and the test construction.

## Introduction

The main goal of this study is to investigate aspects of visual-spatial cognition in athletes and non-athletes using an object-based mental rotation task. Mental rotation is the process of imagining an object when it is rotated away from its original position (Shepard & Metzler, 1971). This ability seems to be important for different professionals, for example, for surgeons, pilots, architects (see Hegarty & Waller, 2005). Furthermore, mental rotation is involved in problem solving (Geary, Saults, Liu, & Hoard, 2000), acquiring mathematical knowledge (Hegarty & Kozhevnikov, 1999), and academic thinking (e.g., Peters, Chisholm, & Laeng, 1995).

In addition to these cognitive skills, motor processes seem to play an important role while solving a mental rotation task. This was first investigated from an experimental point of view in the study of Wexler, Kosslyn, and Berthoz (1998), who showed that if the direction of a mental and a simultaneously conducted manual rotation with a handle were not compatible, reaction times (RTs) were slower and more errors were made than if the direction of both rotations were compatible with each other (cf. Wohlschläger & Wohlschläger, 1998). Furthermore, Meijer and van den Broek (2010) showed a benefit from active exploration in a mental rotation task but only for participants with low visual-spatial abilities. Apart from this study, which relates a motor task of rotating a handle with a mental rotation task, other studies have investigated the effect of a more comprehensive motor activity (viz., physical activity) on mental rotation performance. One such activity that has been investigated is juggling. A positive influence of 3 months of juggling training on a chronometric mental rotation task with cube figures, compared to a control group which did not receive any training, was shown by Jansen, Titze, and Heil, (2009). Moreau, Clerc, Mansy-Dannay, and Guerrin (2012) defined this motor effect in more detail with another type of physical activity. They showed that students who received wrestling training over 10 months outperformed students who received 10 months of running training in mental rotation ability measured from pre- to post-test. Further evidence comes from studies with children who demonstrate reduced motor performance. These studies revealed that motor impaired children also demonstrate an impaired mental rotation performance (Jansen, Schmelter, Kasten, & Heil, 2011; Wiedenbauer & Jansen-Osmann, 2007).

If motor ability or physical activity is related to mental rotation performance, one might argue that people with advanced motor abilities or with a high amount of physical activity should show an enhanced mental rotation performance. This was measured by a higher accuracy rate or a faster RT on a mental rotation test compared to a group which showed less advanced motor abilities and a lower amount of physical activity. This assumption was investigated in a study of Pietsch and Jansen (2012). They found better mental rotation performance in sports and music students compared to a group of students of education science. However, when the time spent practicing, either sports or music was included in the analysis the advantage disappeared, suggesting that the time spent on sport and music activity contributes to the better mental rotation performance. This effect could be explained by different brain adaptations due to the training. For example, Jäncke, Koeneke, Hoppe, Rominger, and Hänggi (2009) have demonstrated that golf experts showed an increase in gray matter in the intraparietal sulcus, a brain area which is involved in mental rotation.

This advanced mental rotation performance in athletes was investigated in detail in several other studies concentrating on specific sport practice. In one study it was shown that two groups of athletes, namely (a) gymnasts who used mental and physical rotations in their practice and (b) athletes whose activities required very little rotation, showed a better mental rotation performance than non-athletes (Ozel, Larue, & Molinaro, 2002). Furthermore, a study revealed that elite athletes who completed daily practice of a combat sport (fencing, judo, and wrestling) showed a higher mental rotation performance than elite runners (Moreau, Mansay-Dannay, Clerc, & Guerrien, 2011). Recently, Jansen, Lehmann, and Van Doren (2012) showed that soccer players demonstrated an improved mental rotation performance (in this case shorter RTs) compared to non-athletes in a chronometric mental rotation task, but only with embodied figures and not with cube figures. This effect was only found for embodied figures and might be explained by the fact that soccer players are trained to recognize the manipulation of bodies in space (the field). Since rotation speed did not differ between soccer-players and non-athletes, the better mental rotation performance might be attributable to more advanced encoding of the stimuli and not to the rotation process itself. Soccer players were chosen to be investigated based on the idea that they are trained by perceiving space and objects from a non-egocentric point of view and thus should be better at object-based transformations. However, this study was limited by the fact that only male soccer players participated and not athletes from other sports or female athletes. Soccer players are trained to perceive objects and to analyze spatial relationships from a non-centered point of view, whereas for example gymnasts are mostly trained in their own body transformation around all three axes (Steggemann, Engbert, & Weigelt, 2011).

The studies mentioned above investigated object-based spatial transformations: Participants had to decide if the two presented (rotated) objects are the same or different. In contrast to the same-different decision task, egocentric transformations require a left-right judgment, for example deciding if a human figure raises the left or the right arm. In one study (Steggemann et al., 2011), rotational experts (e.g., gymnasts) showed a better performance compared to non-experts on left-right judgments with body stimuli. There was no such advantage for object-based transformations using letters (Steggemann et al., 2011). One critical point when looking at these studies is that not only the kind of decision (same-different) but also the kind of stimuli (letters or body figures) were varied.

There are studies which investigated the mental rotation performance in soccer players (Jansen et al., 2012) and which investigated the performance in gymnasts (Jola & Mast, 2005; Steggemann et al., 2011), but no study compared these groups in one experiment. Furthermore, there is no study that investigated the mental rotation performance of athletes in an object-based mental rotation task using a same-different decision task with cube figures and human figures as stimulus material. This experiment strives to close this gap. It is the main goal of this study to investigate the object-based mental rotation performance in male and female soccer players and gymnasts using cube figures and human stimuli (Alexander & Evardone, 2008). Because gender differences favoring males in mental rotation performance are well known in mental rotation tasks (e.g., Voyer, 2011), Gender was considered as a factor. Alexander and Evardone showed that the mental rotation performance with human stimuli was less dependent on gender as compared to the performance with cube figures. This is in line with a study of Amorim, Isableu, and Jarraya (2006) who showed in a chronometric mental rotation test that RT was faster and error rate lower when the abstract cube figures were “embodied” by drawings of humans as rotated cube figures. Thereby the enhancing effect in the study of Alexander and Evardone was made smaller for male compared to women. It might be assumed that the gender difference further diminishes within athletes. In detail, the following hypotheses were investigated:

First, on the basis of the results of the studies of Amorim et al. (2006) and of Alexander and Evardone (2008), it is hypothesized that for all participants, the test performance will be better with human figures compared to cube figures. This is in line with the embodied cognition theory which states that body stimuli elicit embodied processing at a spatial as well as at a motoric level. Second, and again according to the study of Alexander and Evardone, the gender difference in mental rotation will be reduced with human figures. Third, according to the “positive” motor training studies on mental rotation (e.g., Jansen et al., 2012; Pietsch & Jansen, 2012), athletes will show a better mental rotation performance compared to non-athletes in object-based transformation tasks. What has not been considered in research until now is the question whether differences in object-based mental rotation performance will appear between athletes of different sports. We investigated the object-based mental rotation performance in soccer-players and gymnasts to explore this question. As mentioned above, soccer players are trained to perceive objects from a non-centered point of view, whereas gymnasts received a completely different kind of training by the rotation training of their own body rotation. Furthermore, a possible interaction between Gender and Sport Discipline on mental rotation performance must be investigated.

## Method

### Participants

Sixty males (*M*_age_ = 24.11 years, *SD* = 4.84) and sixty females (*M*_age_ = 22.83years, *SD* = 3.38), mostly students, participated in this study. According to their sporting activity, the participants of each gender were divided into the following groups: 40 participants were soccer players, 40 gymnasts, and 40 were non-athletes (see Table 1). The mean number of years spent practicing their respective sport differed between groups, *F*(2, 114) = 55.11, *p* < .001, η^2^ = .49, and genders, *F*(1, 114) = 11.24, *p* = .01, η^2^ = .09. Post-hoc Bonferroni corrected tests showed that both gymnasts and soccer players spent more years practicing sport than non-athletes (*p* < .001). Both groups did not differ from each other in their mean number of year’s athletic activity. Males spent more years practicing their sport (*M* = 10.88, *SD* = 8.04) than females (*M* = 7.7, *SD* = 6.2), *F*(1, 114) = 11.24, *p* <. 01, η^2^ = .09. The mean number of hours per week of sports practice differed between groups, *F*(2, 114) = 115.62, *p* < .001, η^2^ = .67. Post-hoc Bonferroni corrected tests showed that both gymnasts and soccer players spent more hours practicing their sport than non-athletes (*p* < .001), but both athlete groups group did not differ from each other in their mean number of hours per week of practice. Furthermore, for all athletes it was registered if they participated in their sport on a non-competitive or competitive level. There was no difference between both sport groups (*U* = 748.5, *z* = -.572, *ns*). All participants were recruited at sport clubs or at the university and gave their informed consent to participate in this study. None of the participants had solved a mental rotation test before or had been trained in mental rotation.

**Table 1.** Age, Hours, and Years of Sports Practice, Dependent on Group and Gender

	Soccer players		Gymnastics		Non-athletes	
	Males^a^	Females^a^	Males^a^	Females^a^	Males^a^	Females^a^
Age (years)	24,00 (3,24)	23,55 (2,52)	23,8 (6,97)	21,8 (4,42)	24,55 (3,63)	23,15 (2,79)
Cognitive speed^b^	59,97 (14,38)	50,40 (7,78)	58,31 (8,77)	66,17 (22,23)	59,71 (9,81)	60,73 (10,91)
Hours per week	2,95 (0,88)	2,45 (0,60)	2,4 (0,68)	2,65 (1,03)	0,51 (0,50)	0,36 (0,58)
Years of sport	16,15 (3,75)	12,05 (5,95)	13,5 (7,92)	9,2 (4,34)	3,0 (4,72)	1,85 (2,94)

Note. Data shown are means. Standard deviations in parentheses. ^a^ *n* = 20. ^b^ Cognitive speed in seconds.

### Material and procedure

**Figure 1.**
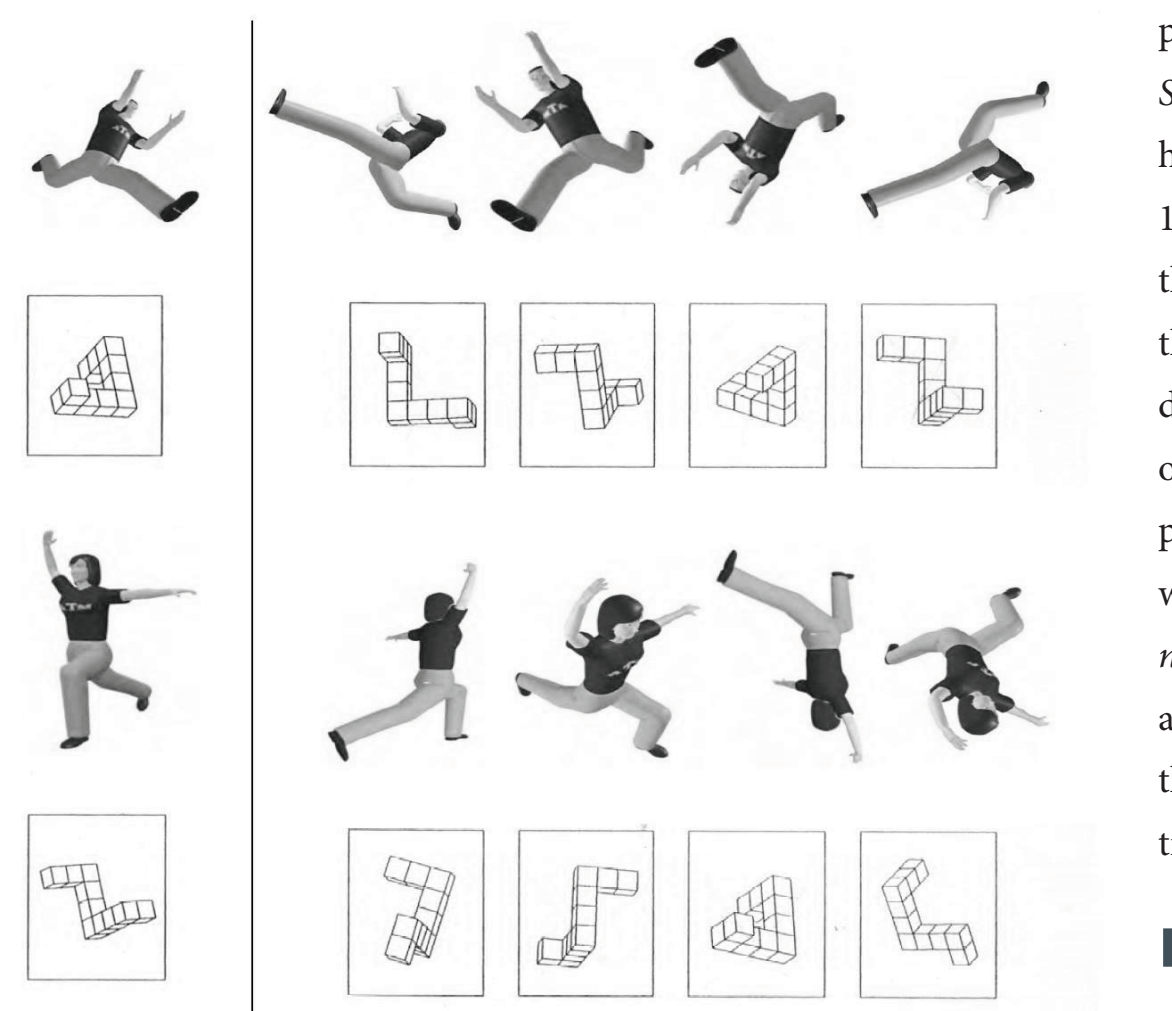
First four sample items from the mental rotation task with human postures and cube figures (Alexander & Evardone, 2008)

A psychometric mental rotation test (MRT) with two different three-dimensional stimulus types, cubes and human figures (Alexander & Evardone, 2008), was solved by all participants (see Figure 1). The psychometric test consisted of 24 items with one target item and four alternatives (two correct and two distracters). Twelve of the items were original items from the MRT by Peters, Laeng, et al. (1995), and 12 items were human figures (six males and six females dressed in identical T-shirts and pants). The 12 original test items (Item 2, 4, 6, 8, 10, 12, 14, 16, 18, 20, and 24) alternated with the 12 test items depicting human figures (Item 1, 3, 5, 7, 9, 11, 13, 15, 17, 19, 21, and 23). For the construction of this test, Alexander and Evardone used the original items of the test of Peters, Laeng, et al. as templates for the spatial orientation of the figure in three-dimensional space and for the serial positioning of correct items and distracters.

The items were presented to the participants on four sheets with six items per sheet. All participants were tested separately or in small groups of up to five participants. Before the test began, training items were given with the solution of these training items at the end of the page. After the training phase, participants were instructed to solve the first 12 items within 3 min, given a short break of 2 min, and then instructed to solve the last 12 items. According to the standard measurement method by Peters, Laeng, et al. (1995), one point was given only if both correct sample stimuli of a target figure were marked correctly. Participants could achieve a maximum of 24 points: 12 points for the human figures items and 12 points for the cube figures items.

Additionally, the cognitive speed was measured for each participant with the ZVT (Zahlen-Verbindungs-Test-Number-Connection-Test; Oswald & Roth, 1987) which is equivalent to the Trail Making Test (Reitan, 1956). In this task, each participant is instructed to connect the numbers 1-90, which are presented on four sheets with an irregular sequence in a matrix of 10 columns and nine rows, in ascending order as fast as possible. The time (seconds) needed for each sheet is measured, and afterwards the time of all four sheets is added and divided by 4. The results of this measurement can be converted into IQ estimations. There is a correlation between ZVT and standard IQ tests of about *r* = .60 to .80 (Vernon, 1993).

### Data analysis

First, a univariate analysis of variance with the dependent variable score, number of correctly solved items on the mental rotation test; and independent variables Gender (male, female), Group (soccer players, gymnasts, and non-athletes), and Stimulus Type (cube figures, human figures) was performed. Secondly, to investigate if possible effects of group and gender on the score can be attributed to differences in group effects, a co-variate analysis was performed. Thereby soccer players,gymnasts, and non-athletes were compared using two variables: (a) hours (hours of sports practice per week) and (b) years (years of actively practicing the relevant sports).

Third, a univariate analysis of variance with the dependent variable cognitive processing speed and the independent variables Gender (male, female) and Group (soccer players, gymnasts, and non-athletes) was performed.

## Results

### Mental rotation

**Figure 2.**
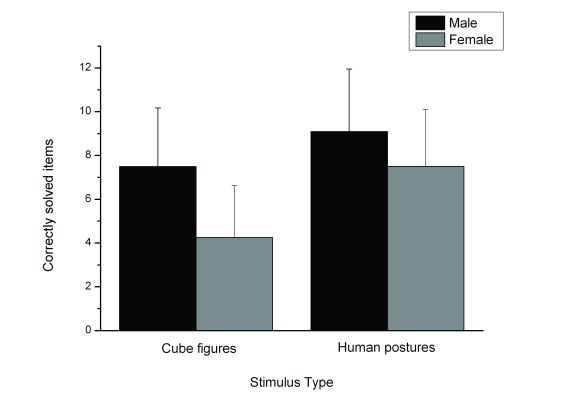
Correctly solved items dependent on stimulus material and gender.

The analysis of variance showed a main effect for the factors Stimulus Type, *F*(1, 114) = 100.26, *p* < .001, η^2^ = .468, and Gender, *F*(1, 114) = 35.23, *p* < .001, η^2^ = .236, as well as a significant interaction between Stimulus Type and Gender, *F*(1, 114) = 11.60, *p* = .001, η^2^ = .092. The performance for human figures (*M* = 8.30, *SD* = 2.83) was better than for cube figures (*M* = 5.88, *SD* = 3.00). Males solved more items correctly (*M* = 16.6, *SD* = 4.92) than females (*M* = 11.78, *SD* = 4.16). The gender difference was higher for cube figures (*d* = 1.78) than for human figures (*d* = 0.82), as shown in Figure 2.

Furthermore, there was a significant main effect for the factor Group, *F*(2, 114) = 3.63, *p* =.03, η^2^ = .060 (gymnasts: *M* = 7.56, *SD* = 2.56;soccer players: *M* = 7.44, *SD* = 2.18; non-athletes: *M* = 6.43, *SD* = 2.79). Bonferroni post-hoc tests revealed that only the difference between gymnasts and non-athletes reached significance (*p* = .04), the difference between soccer players and non-athletes failed to reach significance (*p* = .1). All other interactions were not significant.

To investigate the difference between the performance of soccer-players, gymnasts, and non-athletes, a co-variate analysis was con-ducted including the variables years and hours as co-variates. This analysis was important because both soccer players and gymnasts differed in their amount of practicing sports, measured by years and hours per week, compared to non-athletes. The co-variance analyses showed that only the factor Gender, *F*(1, 112) = 36.46, *p* < .001, η^2^ = .246, and the factor Stimuli, *F*(1, 112) = 11.36, *p* = .001, η^2^ = .09, reached significance, and that there was an interaction between both factors, *F*(1, 112) = 11.75, *p* = .05, η^2^ = .095. However, the effect of the factor Group was no longer significant, *F*(2, 112) = 0.59, *ns*.

**Figure 3.**
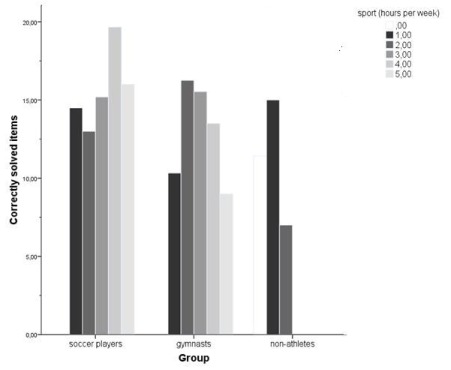
Correctly solved items dependent on sports activity and group.

A correlation analysis separated for each group showed a significant correlation between the mental rotation performance and the amount of practicing sport per week only for soccer players (*r* = .310, *p* = .05, *n* = 40) but not for gymnasts (*r* = -.066, *ns*, *n* = 40) and non-athletes (*r* = .198, *ns*, *n* = 40). There was no correlation in any of the three groups between the mental rotation performance and the years of practicing sports (see Figure 3).

### Cognitive processing speed

The analysis of variance showed no main effects for the factors Group, *F*(2, 114) = 3.00, *ns*, and Gender, *F*(1, 114) = 0.009, *ns*, but a significant interaction between Group and Gender, *F*(1, 38) = 0.98, *ns*. There was no difference between males and females for the non-athletes, *F*(2, 114) = 4.39, *p* < .05, η^2^ = .072, and for the gymnasts, *F*(1, 38) = 2.165, *ns*, but there was a difference favoring females for the soccer players, *F*(1, 38) = 6.85, *p* < .05, η^2^ = .153 (see Table 1). Within the group of soccer players, females (*M* = 50.4 s, *SD* = 7.78) needed less time to solve the ZVT than males (*M* = 59.97 s, *SD* = 14.38).

The main effects and the interaction of the mental rotation analysis did not differ if the cognitive speed was included in the analysis. Furthermore, there was no significant correlation between the performance in the ZVT and the MRT (r = -.14, *ns*).

## Discussion

The results confirmed that the mental rotation performance is higher for human figures compared to cube figures. This may be very well interpreted within the framework of embodied cognition. Spatial embodiment describes the process of mapping one’s body axes onto the stimuli; motor embodiment is the process of imitating the posture using the motor system (Amorim et al., 2006). It seems evident that the human figures facilitate spatial as well as motor embodiment for all participants. This idea is supported by Kessler and Thompson (2016) who point out that looking from a spatial perspective and subsequently using embodied processing allows one to adopt the perspective of the human figures in this study and therefore, the judgment for those postures could be more easily facilitated. The influence of body positions, hand, or arm positions, respectively, on mental rotation performance has also been supported by Ionta, Fourkas, Fiorio and Aglioti (2007) and by Ionta and Blanke (2009).

Furthermore, the results confirmed the second hypothesis that the gender gap is reduced with embodied stimuli. This is in line with the study of Alexander and Evardone (2008) and adds to the literature that gender differences in psychometric mental rotation tests vary according to the test and items construction (Voyer & Hou, 2006). Comparable to the study of Alexander and Evardone, the gender difference in our study was reduced by approximately half. According to Alexander and Evardone, this can be explained by the fact that the human figures promote a holistic strategy, implying that the figure is rotated as a whole. Normally, women are less likely to apply such a strategy (Geiser, Lehmann, & Eid, 2006) but if they are prompted to use it, larger improvements for them compared to males are expected. One point that they do not discuss is whether the items in their test (cube figures vs. human figures) are comparable to each other as it was done with the embodied and non-embodied items in the study of Amorim et al. (2006).

Our third hypothesis, regarding the “motor effect”, could be confirmed. However, post-hoc tests revealed that only gymnasts showed a better mental rotation performance than non-athletes. This “positive effect” did not depend on stimulus type. These results are contradictory to both the results of Steggemann et al. (2011) and of Jola and Mast (2005). Steggemann et al. showed that rotational experts showed no better performance compared to non-experts in same-different judgments with letters as stimulus type. Jola and Mast even found an impaired mental rotation performance in dancers compared to non-dancers within a chronometric mental rotation task using cube figures. Cognitive speed could not explain the better performance of the gymnasts compared to the soccer players, because both groups did not differ in their processing speed.

Furthermore, mental rotation performance did not differ between soccer players and gymnasts depending on the stimulus type. This is quite astonishing because both groups of athletes have a different kind of motor and spatial training. Soccer players are spatially trained in three-dimensional space, learning spatial configurations also from a non-egocentric point of view. Gymnasts have an advanced training experience in real and imagined body transformations in space and around all body axes which could have resulted in a different processing of the abstract and body stimuli. But this idea could not be confirmed by our study. One reason for this might be that the gymnasts have applied a third-person perspective while solving the mental rotation tasks with body stimuli and no first-person perspective by imagining rotating themselves in order to complete this task. To investigate this idea further the analysis of gestures and head movements while solving the tasks with body stimuli might be useful. Imagining oneself in a first-person perspective might induce a movement of some kind with the body.

On the basis of the results of the co-variate analysis it may be argued that the influence of sports practice (per week and per year) is a factor for the better mental rotation performance in the gymnasts. The advantage of the mental rotation performance for gymnasts disappeared when “hours of practicing” and “years of practicing” were included in the analysis. Interestingly, there was no linear relationship between training and mental rotation performance in gymnasts. Instead gymnasts with a moderate training time showed the best mental rotation performance. This shows that the amount of training has to be considered in each study investigating the beneficial of physical activity on any cognitive measurement. The importance of the amount of training has been already mentioned on the basis of a meta-analysis of Colcombe and Kramer (2003) who analyzed the effects of fitness training on the cognitive functions (e.g., executive functions) of older adults. The highest benefit appeared in short and long program durations and moderate session duration.

This study shows that an enhanced physical activity in one sport affects mental rotation performance in an object-based transformation task. It seems that this effect is selective. Only gymnasts who practice rotational movements around the three axes show a better mental rotation performance independent of the type of stimuli. In this study, soccer players did not show a statistically better performance than non-athletes. The mechanisms responsible for these results, some of which are conflicting, are not yet understood. The existing literature differs with respect to many variables: the stimulus type, the kind of test (psychometric vs. chronometric), the dependent variables (accuracy vs. RT vs. mental rotation speed), the kind of motor expertise (rotational experts, etc.), gender, and so on. For example, it has to be considered that psychometric and chronometric tests differ in many aspects: The chronometric test is not time limited and requires often a same-different choice for two items. The psychometric test in contrast to that is often time limited and, in this case, is a two-out-of-four alternatives choice task. Additionally, it might be that the perspective in chronometric tests is more easily perceived than in psychometric tests.

This study is limited by the fact that only the performance of two different types of athletes was investigated and that both did not only differ in their spatial and motor training but maybe also in the effort that is associated with each type of sport. Furthermore, the presentations of the human figures and cube figures have to be controlled for difficulty (cf. Doyle & Voyer, 2013). The result that female soccer players had a better processing speed than males was not the focus of our study and deserves attention in further studies.

To conclude, this study highlights that while considering all mental rotation studies, the conclusions made regarding positive or negative effects of enhanced physical activity should only be drawn carefully, keeping in mind the experimental situation.
